# Persistent Pulmonary Interstitial Emphysema With Respiratory Infection: A Clinicopathological Analysis of Six Cases and Detection of Infectious Pathogens by Metagenomic Next-Generation Sequencing (mNGS)

**DOI:** 10.3389/fped.2022.836276

**Published:** 2022-04-07

**Authors:** Ping Zhou, Weiya Wang, Yiyun Fu, Ying Zhang, Zuoyu Liang, Yuan Tang, Lili Jiang

**Affiliations:** Department of Pathology, West China Hospital, Sichuan University, Chengdu, China

**Keywords:** congenital thoracic malformations, pulmonary interstitial emphysema, respiratory infection, infants, metagenomic next-generation sequencing

## Abstract

**Background:**

Persistent pulmonary interstitial emphysema (PPIE) is known to be related to mechanical ventilation and preterm. However, PPIE is also reported rarely in non-ventilated and full-term infants. Its relationship with respiratory infection is rarely reported in the literature. PPIE is difficult to diagnose and always mimics other congenital thoracic malformations (CTMs), such as congenital cystic adenomatoid malformation (CCAM).

**Objective:**

The objective of this study was to evaluate clinicopathological and radiographic features of PPIE with respiratory infection and to detect the possible infectious pathogens.

**Methods:**

From January 2011 to December 2019, six cases were confirmed pathologically with PPIE from a large cohort of 477 resected CTMs in West China Hospital of Sichuan University. Clinical and radiographic features were obtained from patients' medical records and follow-up. The present study aimed to analyze clinicopathological and radiographic features and to detect the infectious pathogens by metagenomic next-generation sequencing (mNGS).

**Results:**

The six PPIE cases included four girls and two boys, ranging from 2 months to 5 years; 100% (5/5) of the available cases were full-term and without mechanical ventilation. CCAM were suspected in 66.7% (4/6) patients; 66.7% (4/6) cases affected a single lobe, and 33.3% (2/6) cases affected both lung lobes. Clinically, all six PPIEs were presented with symptoms of respiratory infection and diagnosed with pneumonia. All six patients were treated by surgery after anti-infective treatment. The pathologic characteristics showed lung cysts with variable size along the bronchovascular bundles, the cysts had a discontinuous fibrotic wall with a smooth inner surface lined with uninucleated and/or multinucleated macrophages. *Streptococcus pneumoniae* was detected in patient No. 1. Human beta-herpesvirus 5 was detected in patient No. 2. *Neisseria mucosa, Neisseria sicca, Prevotella melaninogenica, Prevotella histicola*, and *Fusobacterium nucleatum* were detected in patient No. 5, and no infectious pathogen was detected in 50% (3/6, No. 3, No. 4, and No. 6) of cases.

**Conclusion:**

Six rare cases of PPIE with respiratory infection were treated by surgery after anti-infective treatment. All five available cases were full-term infants without mechanical ventilation. The histological characteristics of PPIE were the wall of cysts composed of a thin layer of discontinuous fibrous tissue and lined with uninucleated or/and multinucleated macrophages.

## Introduction

With routine prenatal ultrasound scans performed, more and more congenital thoracic malformations (CTMs) are diagnosed in infants (1, 2), but the incidence is rare, which is present in 1 per 10,000–35,000 births (3). The differential diagnosis of CTMs contains congenital cystic adenomatoid malformation (CCAM), pulmonary sequestration (PS), bronchogenic cyst, congenital lobar emphysema (CLE), persistent pulmonary interstitial emphysema (PPIE), and so on.

Pulmonary interstitial emphysema (PIE) is a rare cystic disease of infants (4). PIE is an air leak syndrome, characterized by gas dissecting pulmonary interstitium along the bronchovascular bundles. There are three clinical types of PIE, including acute IPE, local persistent PIE (LPPIE), and diffuse persistent PIE (DPPIE) (5, 6). Acute IPE is <7 days in duration, diffuse persistent PIE is observed when small cysts are noted in all lobes of the lung, and local persistent PIE affects only one lobe (7). Chest computed tomography (CT) scan sometimes was limited to diagnose PIE. CT showed cystic lung lesions mimicking CCAM in the postnatal period (8). The definitive diagnosis is histological. A histological diagnosis of PIE was established through the wall of cysts composed of a thin layer of discontinuous fibrous tissue and lined with uninucleated or/and multinucleated macrophages (9, 10).

PIE is known to be related to mechanical ventilation and preterm (11, 12). However, it is also reported rarely in both non-ventilated and full-term infants (13, 14). Pursnani et al. (14) showed a 3-month-old infant with LPPIE who had no history of respiratory distress syndrome (RDS) and mechanical ventilation; the patient had a medical history of viral pneumonia 1 month prior to surgery, indicating respiratory infection may be related to PPIE.

However, there were just a few reports of PPIE with respiratory infection (13–22), and possible infectious pathogens were still unclear. With the development of molecular methods of identification, the metagenomic next-generation sequencing (mNGS) is a novel, rapid, simple, and convenient approach to the clinical identification of infectious diseases.

In the present study, we report six rare cases of PPIE with respiratory infection, followed by successfully surgical treatment. To our best knowledge, it is the first time to detect the possible infectious pathogens in PPIE by using mNGS.

## Materials and Methods

### Case Series and Clinicopathological Features

From January 2011 to December 2019, 477 resected CTMs in West China Hospital of Sichuan University were retrospectively rescreened independently by two pathologists (P Zhou and LL Jiang). According to the histological criteria of PIE, six PPIEs were enrolled in the present study.

Clinical and radiographic features were obtained from patients' medical records and follow-up. We retrospectively collected age, sex, term, mechanical ventilation, prenatal ultrasound, clinical features, radiographic features, affected sites, and the diameter of the cystic lesions.

### Special Stain

Special stains (acid fast stain, Gomori's methenamine silver staining, and Giemsa) and TB-PCR (Qiagen) were carried out for all cases according to the manufacturer's protocol.

### DNA Extraction, Library Construction, and Sequencing

DNA was extracted from available blocks with the TIANamp Micro DNA Kit (DP316, Tiangen Biotech) following the manufacturer's protocol. We constructed DNA libraries according to the standard protocol through end-repaired adapter added overnight and by applying polymerase chain reaction amplification to the extracted DNA. To measure the adapters before sequencing, quality control was carried out using a bioanalyzer (Agilent 2100, Agilent Technologies, Santa Clara, CA, USA) combined with quantitative PCR. DNA sequencing was then performed with the BGISEQ-100 platform.

### Data Processing and Analysis

High-quality sequencing data were generated after removing low-quality, low-complexity, and shorter reads. The data mapped to the human reference genome (hg19) were excluded using a powerful alignment tool called Burrows–Wheeler Alignment to eliminate the effect of the human sequences. The database used for the present study includes 6,350 bacteria, 1,798 viruses, 1,064 fungi, and 234 parasites, which all relate to human disease. Finally, the mapped data were processed after filtering out duplicate reads for advanced analysis. The SoapCoverage from the SOAP website was used to calculate the sequence depth and genomic coverage for each species.

## Results

### Clinical Characteristics

From January 2011 to December 2019, 477 CTMs were retrospectively rescreened in West China Hospital of Sichuan University. CTMs were consisted of congenital cystic adenomatoid malformation (CCAM) (286, 60%), pulmonary sequestration (PS) (143, 30%), bronchogenic cyst (29, 6.1%), congenital lobar emphysema (CLE) (13, 2.7%), and persistent pulmonary interstitial emphysema (PPIE) (6, 1.3%).

All six PPIEs were treated by surgery after suggested anti-infective treatment therapy. The clinical characteristics of the six patients are shown in Table 1. There were four girls and two boys, ranging from 2 months to 5 years old. We collected the follow-up data of all patients except for No. 4. Patient No. 4 had the wrong phone number. The other available patients (5/5, 100%) were all full-term without mechanical ventilation. Clinically, all six cases of PPIE were presented with symptoms of respiratory infection and diagnosed with pneumonia. The common symptoms of the patients were cough, fever, and expectoration. Before surgical treatment, all six patients received suggested anti-infective treatment therapy. The cystic lesions were located at a single lobe among 66.7% (4/6) patients, who were identified with local PPIE, and both lung lobes among 33.3% (2/6) patients, who were identified with diffuse PPIE.

**Table 1:** Clinical features of the six cases with PPIE.

**No**.	**Sex**	**Age**	**Other defects**	**Ultrasound finding**	**Term**	**Mechanical ventilation**	**CT diagnosis**	**Clinical magnification**	**Affected site**	**Diameter (cm)**
1	M	3 y	None	None	Full term	None	CCAM	Pneumonia for 10 days	Right lower lobe	2.5
2	F	3 y	None	None	Full term	None	CCAM	Pneumonia for 1 month	Right upper lobe	1.8
3	F	1 y	None	None	Full term	None	CCAM	Pneumonia for 1 month	Both lower lobes	5.6
4	M	11 m	None	None	NA	NA	Cystic lesion	Recurrent pneumonia for 7 months	Right lower lobe	3.8
5	F	5 y	Ventricular septal defect	None	Full term	None	Cystic lesion	Recurrent pneumonia for 3 months	Both lung lobes	-
6	F	2 m	None	None	Full term	None	CCAM	Pneumonia for about 20 days	Right lower lobe	2.6

*F, female; M, male; y, years; m, months; CCAM, congenital cystic adenomatoid malformation; NA, not available*.

Besides this, patient No. 5 suffered the disease Langerhans cell histiocytosis (LCH) in the left submandibular lymph node for 1 year. She was asymptomatic when she received the suggested chemotherapy treatment of four courses; she suffered recurrent pneumonia for 3 months, and the routine examination of the chest CT found the diffuse cystic lesions in both lung lobes. In addition, she had the defect of ventricular septal defect.

### Imaging Findings

**Figure 1:**
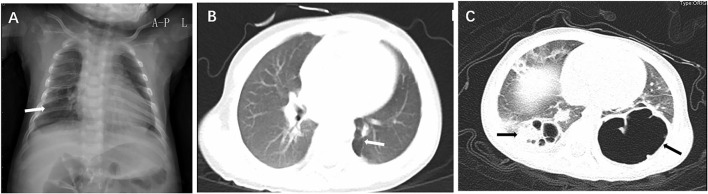
Chest X-ray showed translucency of the right lower lobe (**A**, arrow); CT scan of the chest showed a single cyst at the left lower lobe (**B**, arrow) and bilateral cysts in left and right lower lobes (**C**, arrows).

Prenatal ultrasound did not find any lesion in all six patients. Chest X-ray showed translucency of the affected site (Figure 1A). Chest CT scan of all the cases showed cystic lesions. According to the CT findings, CCAM was suspected in 66.7% (4/6 cases). The radiographic patterns of PIPE ranged from a cystic lesion or multicystic lesion of one or more lobes to a diffuse multicystic involvement of all lobes. CT scan of the chest showed a single cyst (Figure 1B) and bilateral cysts in both lungs (Figure 1C). The affected site was the right lower lobe in 50% (3/6 patients), the right upper lobe in 16.7% (1/6 patient). The local multicystic pattern in 66.7% (4/6) PPIE cases included a wide range of sizes for the cystic lesions (1.8–3.8 cm). There are 33.3% (2/6 patients) affected in both lung lobes.

### Pathological Examination

All the patients were treated by surgery after suggested conventional anti-infective treatment. The resected affected site was the right lower lobe in 50% (3/6 patients), the right upper lobe in 16.7% (1/6 patient). There are 33.3% (2/6 patients) (No. 3 and No. 5) affected in both lung lobes; the resection of the right lower lobe was made in patient No. 3, and the resection of the left lobe was made in patient No. 5.

The gross specimens showed multiloculated cysts with variable size within the pulmonary parenchymal, and the cysts had a smooth inner surface. Some cysts contained a small amount of clear fluid.

**Figure 2:**
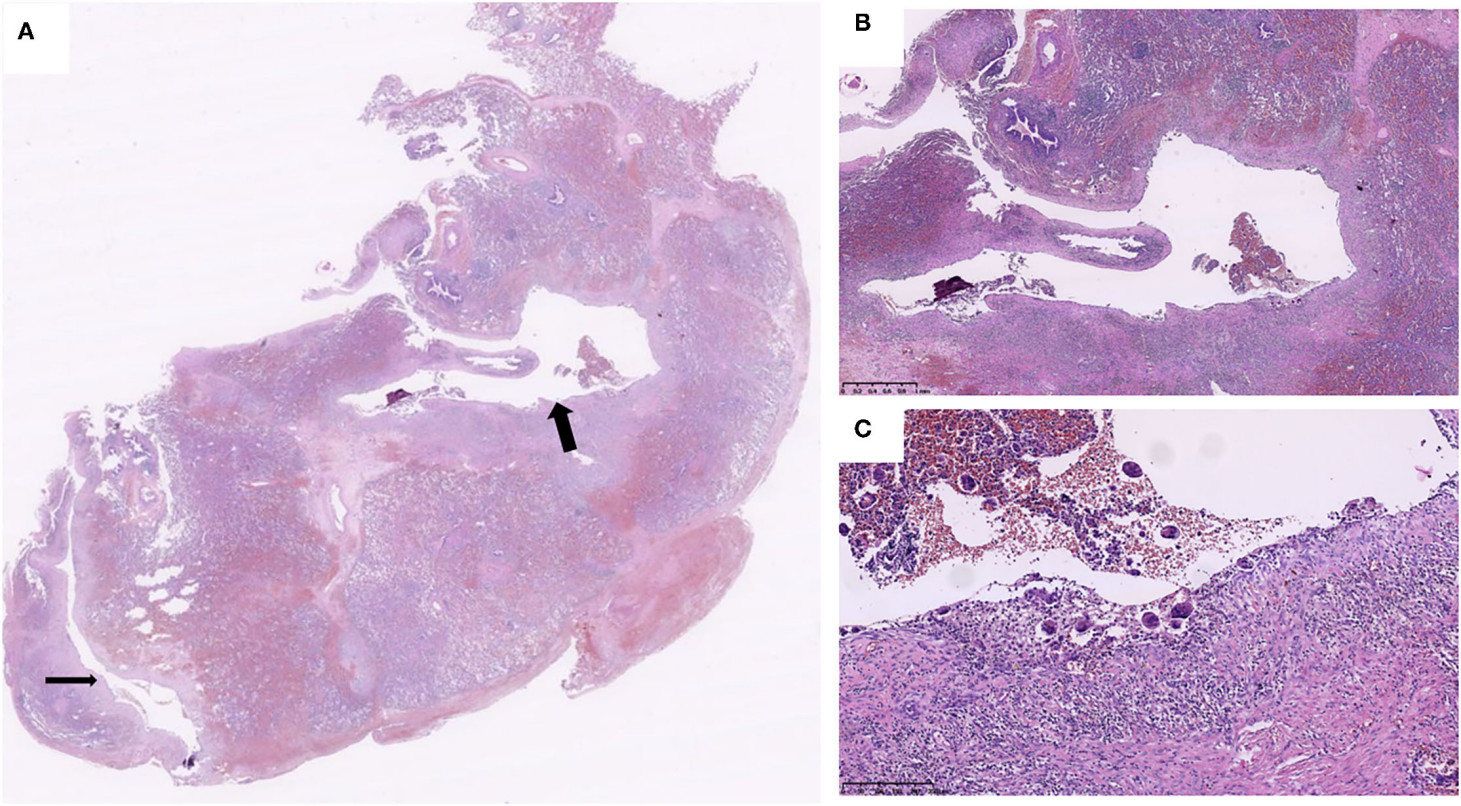
Two air cysts (arrows) were around the bronchovascular bundles **(A)**, and the cystic walls primarily consisted of connective tissue. The cystic walls are consisted of discontinuous connective tissue and lined with uninucleated and multinucleated macrophages **(B,C)** (Case 2).

**Figure 3:**
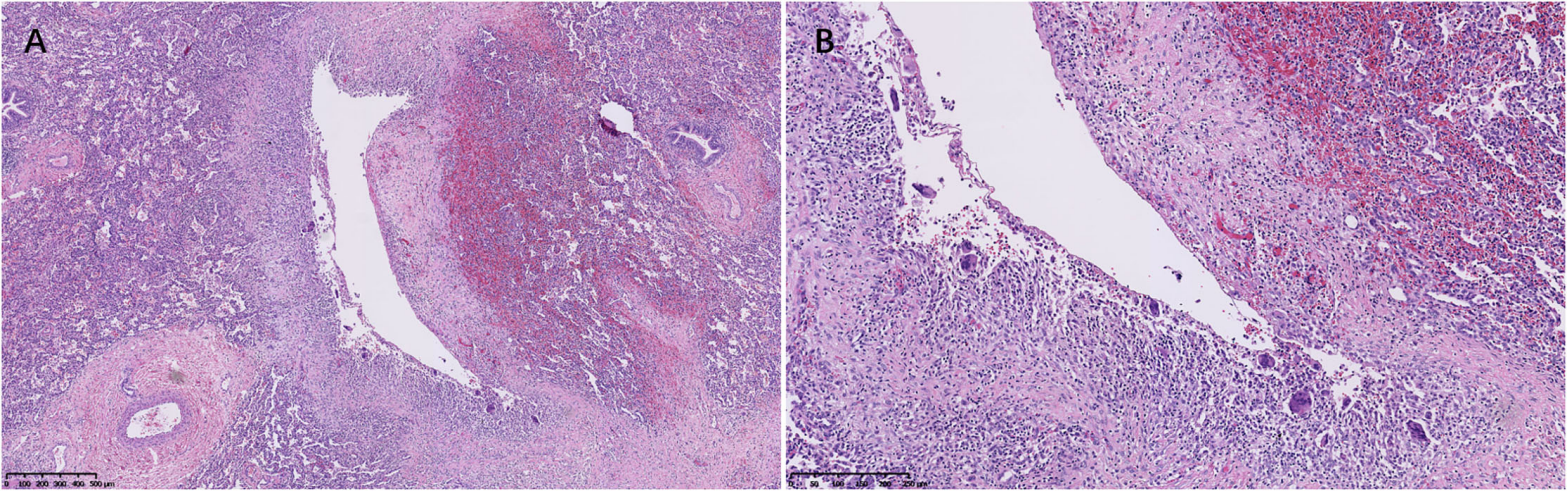
An air cyst was around the bronchovascular bundles **(A)**, and the cystic walls consisted of connective tissue lined with uninucleated and multinucleated macrophages **(B)** (Case 3).

Microscopically, histological observation found that the walls of the cysts were adjacent to interlobular septa or bronchovascular bundles, the wall of cysts was composed of a thin layer of fibrous tissue, and the thin band of fibrous tissue was discontinuous. Small collections of uninucleated and multinucleated macrophages lined the surface of the main cysts (Figures 2, 3). The giant cells contained from 2 to 40 centrally placed nuclears.

The adjacent parenchymal surrounding the cysts showed mild to marked atelectasis and inflammatory cells infiltrates in all cases (Figures 2, 3), containing histocytes, lymphocytes, plasma cells, and neutrophils, indicating there was inflammation along the cysts. Mucosal edema, cellular debris of the bronchi, mucus, and inflammatory exudates of the bronchi can be seen.

### Special Stain and TB-PCR

Special stains (acid fast stain, Gomori's methenamine silver staining, and Giemsa) and TB-PCR (Qiagen) were carried out for all six cases according to the manufacturer's protocol. None was seen in the case series, indicating it did not contain any identifiable organism or foreign material in all six cases.

### Infectious Pathogens Detected by mNGS

The next-generation sequencing was performed from blocks of resected lung samples for each patient. In the current study, mNGS successfully identified the infectious pathogens in all patients, and the pathogens detected are shown in Table 2. Before surgical treatment, all the six patients received suggested anti-infective treatment therapy. Therefore, infectious pathogens were detected in three cases. *Streptococcus pneumonia* (specific reads: 84) was detected in patient No. 1. Human beta-herpesvirus 5 (specific reads: 20) was detected in patient No. 2. *Neisseria mucosa* (specific reads: 587), *Neisseria sicca* (specific reads: 247), *Prevotel lamelaninogenica* (specific reads: 258), and *Prevotella histicola* (specific reads: 174) and *Fusobacterium nucleatum* (specific reads: 239) were detected in patient No. 5. No infectious pathogen was detected in 50% (3/6) cases (patients No. 3, No. 4, and No. 6).

**Table 2:** Infectious pathogens and metagenomic next-generation sequencing (mNGS) results.

**Case**.	**Sex**	**Age**	**mNGS results**	**Specific reads (***n***)**
No.1	M	3 y	*Streptococcus pneumoniae*	84
No.2	F	3 y	Human beta-herpesvirus 5	20
No.3	F	1 y	Not found	
No.4	M	11 m	Not found	
No.5	F	5 y	*Neisseria mucosa*	587
			*Neisseria sicca*	247
			*Prevotella melaninogenica*	258
			*Prevotella histicola*	174
			*Fusobacterium nucleatum*	239
No.6	F	2 m	Not found	

*F, female; M, male; y, years; m, months*.

## Discussion

The pathophysiology of PIE is a result of air leakage into the interstitium from alveolus due to disruption of the alveolar wall basement membrane, which may dissect along the bronchovascular bundles and radiate outward to the periphery of the lung, mediastinum, and pericardium. PIE included local persistent PIE, acute PIE, and diffuse persistent PIE (5, 6). Diffuse persistent PIE is observed when small cysts are noted in all lobes of the lung (7), and acute IPE is <7 days in duration.

PIE is a rare condition that commonly affects newborn infants with a history of prematurity with positive pressure mechanical ventilation (11, 12). However, it is also reported rarely in both unventilated and full-term infants (13, 14). There are only a few cases reported for PIE developing in unventilated neonates (5, 13, 14, 21, 23–25). In our study, six rare PPIEs from a large cohort of 477 resected CTMs and five available cases were full-term infants without mechanical ventilation.

Chest CT sometimes may show air surrounding the bronchovascular bundles in patients with PIE (5). A multi-institutional study found that about 82% patients with PPIE had the characteristic CT findings with central lines and dots surrounded by radiolucency (26). Chest CT is not an effective diagnostic tool for PIE presenting as multiple cysts with various sizes in one or more lobes of the lung (15). In particular, CT showed cystic lung lesions mimicking CCAM (8). When a patient does not have classical CT features, PIE should be differentiated from other cystic lung lesions, including CCAM, CLE, bronchogenic cyst, cystic lymphangioma, and so on. According to the CT findings and clinical features, CCAM was suspected in 66.7% (4/6 cases) in the present study. Besides this, there was a rare case in which prenatal ultrasound found cystic lesions in the previous literature (27). Messineo et al. reports a male infant suffering from type I CCAM at 20 weeks of gestation with ultrasound scanning, which was diagnosed with PIE after surgery (27). Prenatal ultrasound did not find any lesions in the lungs of six patients with prenatal ultrasound examination in the present study, which indicates that the PPIE may be formed after birth.

Persistent PIE is pathologically characterized by irregularly shaped and multiloculated cysts of various sizes along the bronchovascular bundle. The cysts are air-filled spaces in the parenchymal and composed of a thin band of fibrous connective tissue. The degree of the fibrosis may vary the duration of PIE. The uninucleated and/or multinucleated macrophages lining the cyst walls are the typical pathological features (9, 10). The typical features of air cysts surrounding the bronchovascular bundles with fibrous tissue lining the uninucleated and/or multinucleated macrophages were both demonstrated among all six cases in our present study.

The differential pathological diagnosis was made with other CTMs, such as CCAM, PS, bronchogenic cyst, CLE, and so on. CCAM is characterized by a lack of communication between the lesion and the tracheobronchial tree and a proliferation of irregularly dilated terminal bronchiole-like structures (28). PS is characterized with non-functioning lung tissue that receives systemic arterial blood supply and does not communicate with the adjacent tracheobronchial tree (29). Bronchogenic cyst is lined with pseudostratified columnar respiratory epithelium, cartilage plate, smooth muscle, and bronchial glands (30). CLE showed lobar hyperinflation with overdistention of normally formed alveoli and without destruction of alveolar walls, aspiration pneumonia, infection, and proximal bronchial obstruction (31); 477 resected CTMs consisted of CCAM (60%), PS (30%), bronchogenic cyst (6.1%), CLE (2.7%), and PIE (1.3%) in the present study.

A standard treatment strategy for PIE has not yet been established. The critical treatment is to be able to maintain sufficient gas exchange (4). Surgical resection of PIE is controversial, and several studies advocate a conservative medical approach (26, 32). A conservative medical approach needs close clinical and radiological monitoring. When there is difficulty in making the diagnosis, the absence of classical CT features, and non-surgical options failed or progressive syndrome, or severe complications, surgery should be considered (33). Jassal et al. report a case that illustrates that extensive bilateral PPIE associated with a persistent pneumomediastinum can resolve spontaneously, thus demonstrating that conservative management without surgical intervention may be appropriate for some children (32). Infants with PPIE and weighing <1,000 g are at significant risk of mortality and associated morbidity of PPIE (34).

The mechanism of production of PIE is the disruption of the alveolar wall basement membrane with subsequent dissection of air into the interstitial space. Given et al. (35) show that PIE in bronchiolitis is thought to occur secondary to inflammation, leading to mucosal edema, increased secretion, and cellular debris, resulting in expiratory obstruction of the small airways; the resulting check-valve effect leads to hyperinflation then alveolar rupture. Another study (13) demonstrates that pneumonia may contribute to the development of pulmonary air leaks by at least three mechanisms: first, air trapping from mechanical or check-valve obstruction within the bronchi by mucus and inflammatory exudates; second, reduced strength or direct disruption of the alveolar lining from parenchymal inflammation or necrosis as commonly seen in necrotizing pneumonia; and third, decreasing lung compliance.

There was obvious inflammatory reaction in the present six rare cases. There are few reports of pulmonary air leakage with respiratory infection. There were just 10 previous articles including 13 patients with PPIE with respiratory infection from a PubMed search (13–22). Review of published literature of PIE with respiratory infection is shown in Table 3. Gala (22) did not mention the sex of the case, so there were seven females and five males, ranged from birth to 87 years. Only 46.2% (6/13) patients with PPIE were treated with surgery. 66.7% (8/12) were full-term, 33.3% (4/12) were preterm. 46.2% (6/13) patients had mechanical ventilation. There was pulmonary air leakage, pneumothorax (5/13 cases), pneumomediastinum (2/13 cases), and mediastinal shift (3/12 cases). According to the previously reported PIE patients with respiratory infection, there were nine patients without certain infectious pathogens reported (13–17, 21, 22). Two patients were infected by respiratory syncytial virus (RSV), and the two were infected by *Candida albicans* (18) and *Staphylococcus aureus* (16). In our study, the common symptoms of the patients were cough, fever, and expectoration. Special stains (acid fast stain, Gomori's methenamine silver staining, and Giemsa) and TB-PCR did not find any identifiable organism or foreign material in our study. No infectious pathogen was detected in 50% (3/6) cases with pneumonia prior to surgery, which may be associated with clinical infectious symptoms being controlled with conventional anti-infective treatment before surgery. *Streptococcus pneumonia* was detected in patient No. 1, whose infectious symptoms were present during surgery. *Streptococcus pneumonia* is a significant human pathogen and a leading cause of bacterial pneumonia in children (36), and *Streptococcus pneumoniae* is a frequent cause of severe community-acquired pneumonia among children in Beijing of China (37). Human beta-herpesvirus 5 (HHV-5) (specific reads *n* = 20) was detected in patient No. 2 without immunodeficiency. *Neisseria mucosa, Neisseria sicca, Prevotel lamelaninogenica, Prevotella histicola*, and *Fusobacterium nucleatum* were detected in patient No. 5. Patient No. 5 has suffered the disease Langerhans cell histiocytosis (LCH) in the left submandibular lymph node for 1 year and has received the suggested chemotherapy treatment of four courses, and then she suffered recurrent pneumonia for 3 months, the detected infectious pathogens may be associated with respiratory infection after chemotherapy treatment for LCH. *Neisseria mucosa* and *Neisseria sicca* are known as common commensals of the upper respiratory tract (38), however, which sometimes are associated with respiratory diseases. Previous studies show that *Neisseria mucosa* and *Neisseria sicca* are consistent with respiratory microbiome from pediatric tracheostomy tubes without granulomas (39), and *Neisseria mucosa* caused pulmonary coin lesion in a child with chronic granulomatous disease (40). A case of spontaneous pulmonary abscess with cavitation caused by *Neisseria mucosa* in a chronically neutropenic child is reported (41).

**Table 3:** Review of published literatures of PPIE with respiratory infection.

**References**	**Cases**	**Sex**	**Age**	**Term**	**Mechanical ventilation**	**Pneumothorax**	**Mediastinal shift**	**Pneumo** **mediastinum**	**Infectious pathogen**	**Affected site**	**Surgical resection**
Toledo Del Castillo et al. (20)	1	M	18 d	Full-term	Yes	No	Yes	No	RSV	Left lung	No
Aiyoshi et al. (19)	1	F	22 m	Full-term	Yes	Yes	No	Yes	RSV	Right upper lobe	No
Gala (22)	1	NA	At birth	Preterm	Yes	No	No	No	Not reported	Left lung	No
Sherren and Jovaisa (21)	1	F	87 y	NA	Yes	Yes	No	No	Not reported	Both lung lobes	No
Lee and Im (13)	1	F	6 w	Full-term	No	Yes	NA	Yes	Not reported	Right upper and middle lobe	No
Pursnani et al. (14)	1	M	3 m	Full-term	No	Yes	No	No	Not reported	Right upper and middle boles	Yes
Crosswell and Stewart (17)	1	M	At birth	Preterm	No	No	No	No	Not reported	Left lower lobe	No
Yao et al. (18)	1	F	9 w	Preterm	Yes	Yes	No	No	*Candida albicans*	Left upper lobe	Yes
O'Donovan et al. (16)	1	F	At birth	Preterm	Yes	No	Yes	No	*Staphylococcus aureus*	Right lung	No
Cohen et al. (15)	4	2F and 2M	45 d to 2 y	Full-term	No	No	1 with mild deviation	No	Not reported	2 right upper lobe, 1 right lower lobe and 1 left upper lobe	Yes

*F, female; M, male; y, years; m, months; w, weeks; NA, not available; RSV, respiratory syncytial virus*.

## Conclusion

Six rare cases of PPIE with respiratory infection were treated by surgery after anti-infective treatment. All five available cases were full-term infants without mechanical ventilation. The diagnoses of PPIE are based on characteristic radiographic imaging and histopathology. The histological characteristics of PPIE were the wall of cysts composed of a thin layer of discontinuous fibrous tissue and lined with uninucleated or/and multinucleated macrophages.

## Data Availability Statement

The datasets presented in this study can be found in online repositories. The names of the repository/repositories and accession number(s) can be found below: DNA Data Bank of Japan (DDBJ), and BioSample Submission ID: SSUB020649.

## Ethics Statement

Ethics approval was obtained from the respective Ethics Committees of West China Hospital, Sichuan University, China (No. 2020892). Written informed consent from the participants' legal guardian/next of kin was not required to participate in this study in accordance with the national legislation and the institutional requirements. Written informed consent was not obtained from the minor(s)' legal guardian/next of kin for the publication of any potentially identifiable images or data included in this article.

## Author Contributions

PZ collected data, analysis and drafted the initial manuscript, and reviewed and revised the manuscript. WW and YF performed data analysis, drafted, and revised the manuscript. YT and LJ reviewed and revised the manuscript. YZ and ZL performed data analysis. All authors read and approved the final manuscript.

## Funding

This work was supported by Sichuan Province Science and Technology Support Program (Grant Number 2020YFS0275) and 1.3.5 Project for Disciplines of Excellence-Clinical Research Incubation Project, West China Hospital, Sichuan University (No. 2019HXFH002).

## Conflict of Interest

The authors declare that the research was conducted in the absence of any commercial or financial relationships that could be construed as a potential conflict of interest.

## Publisher's Note

All claims expressed in this article are solely those of the authors and do not necessarily represent those of their affiliated organizations, or those of the publisher, the editors and the reviewers. Any product that may be evaluated in this article, or claim that may be made by its manufacturer, is not guaranteed or endorsed by the publisher.

## Abbreviations

CTM: congenital thoracic malformations
PIE: Pulmonary interstitial emphysema
PPIE: Persistent pulmonary interstitial emphysema
CT: Computed tomography
mNGS: metagenomic next-generation sequencing
CCAM: congenital cystic adenomatoid malformation
PS: pulmonary sequestration
CLE: congenital lobar emphysema.
